# Antidiabetic Effects of *Aronia melanocarpa* and Its Other Therapeutic Properties

**DOI:** 10.3389/fnut.2017.00053

**Published:** 2017-11-06

**Authors:** Ines Banjari, Andreja Misir, Katarina Šavikin, Stela Jokić, Maja Molnar, H. K. S. De Zoysa, Viduranga Y. Waisundara

**Affiliations:** ^1^Faculty of Food Technology Osijek, Josip Juraj Strossmayer University of Osijek, Osijek, Croatia; ^2^Institute for medicinal plants research “Dr Josif Pančić”, Belgrade, Serbia; ^3^Department of Food Technology, Faculty of Technology, Rajarata University of Sri Lanka, Mihintale, Sri Lanka

**Keywords:** *Aronia melanocarpa*, cardiovascular disease, diabetes, oxidative stress, phenolic compounds

## Abstract

Diabetes is a global pandemic which warrants urgent attention due to its rising prevalence and economic burden. Thus, many alternative therapies are being researched for antidiabetic properties, given the inefficacy of current medicinal treatments. From this perspective, *Aronia melanocarpa* or black chokeberry has been investigated for its therapeutic properties in many studies, especially for its ability to combat hyperglycemia-induced oxidative stress and the macrovascular complications of diabetes including cardiovascular disease. Though *A. melanocarpa* is native to the eastern areas of North America, it has been planted extensively in Europe and Asia as well. Several *in vivo* studies have displayed the antioxidant properties of *A. melanocarpa* berry juice and plant extract in rat models where oxidative stress markers were observed to have significant reductions. Some of the potent bioactive compounds present in the fruits and other parts of the plant were identified as (−)-epicatechin, chlorogenic acid, neochlorogenic acid, and cyanidin-3-galactoside. Overall, *A. melanocarpa* could be considered a good source of antioxidants which is effective in combating hyperglycemia-induced oxidative stress.

## Introduction

Diabetes mellitus is a metabolic disorder of the endocrine system and is currently considered a global pandemic. The disease is prevalent in all parts of the world and is especially on the rise in developing and newly developed nations. Individuals with diabetes are not able of producing or properly utilizing insulin in the body, thus resulting in elevated glucose levels. The cause of diabetes is multi-faceted, although both genetic and environmental factors such as obesity and lack of exercise appear to play critical roles (1–3). Ethnic and racial differences have also been found to contribute to the prevalence of diabetes, especially among heterogeneous populations within the same area (1). Additionally, scientific evidence leads to the belief that in the presence of a particular genetic predisposition, environmental factors trigger the development of diabetes. On the other hand, in type 2 diabetes mellitus, a chronic exposure of βTC3 cells to high glucose levels has been known to result in glucose toxicity *via* increased oxidative stress (4). The injury caused by hyperglycemia-induced oxidative stress can affect all organs. A growing amount of evidence indicates that the consumption of plant foods is correlated with a lower risk from development of oxidative stress-related diseases, especially diabetes (5). Although there are a number of antidiabetic medications, no single marketed drug has succeeded in lowering hyperglycemia-induced oxidative stress. Most therapies target at lowering HbA1c. However, these drugs—when used in combination with other pharmaceutical agents—tend to lose much of their efficacy after continuous usage (6). Weight gain is the primary side-effect of many of the antidiabetic therapies such as sulfonylureas, α-glucosidase inhibitors, and thiazolidinediones. Because many diabetic patients are already obese, this side-effect is particularly undesirable. When it comes to a point where oral therapies can no longer adequately control blood sugar, the only remaining option is injectable insulin therapy. Nevertheless, none of these interventions adequately address oxidative stress, and therefore, the complications arising from the disease condition worsen. As a result, there is an existing need for novel therapies, and more importantly, dietary interventions and changes in the lifestyle to provide both enhanced benefits for diabetic patients. The purpose of this mini review is to serve as an updated summary of the antidiabetic effects of *A. melanocarpa* and its applications in other disease conditions. Several recent studies focused on *A. melanocarpa* potential in this context. Figure S1 in Supplementary Material summarizes all the disease conditions for which *A. melanocarpa* has been used to date (including its traditional medicinal applications) and has been proven effective.

## Antidiabetic Effects of *A. melanocarpa*

From the perspective of oxidative stress, plant-based products which are rich in anthocyanins have been shown to exhibit a plethora of pharmacological properties, such as anti-inflammatory, antitumor, and antioxidant activities. These properties have scientifically displayed beneficial effects for mitigating hyperglycemia-induced oxidative stress and its resulting complications (7–9). *A. melanocarpa* (Michx.) Elliott or black chokeberry is a fruit/plant which has been extensively investigated for its antidiabetic properties. Images of this plant are shown in Figure S2A–C in Supplementary Material and its major bioactive compounds in Figure S2D in Supplementary Material. Due to the astringent taste of these fruits and their smell of bitter-almonds, pure *A. melanocarpa* products are not particularly popular among many consumers, although they have been documented as a “functional food” in Russia since the 1940s (10).

*Aronia melanocarpa* preparations are sometimes consumed as a complementary and alternative therapy for conditions such as achlorhydria, avitaminoses, convalescence, and hemorrhoids (11). High anthocyanin contents in *A. melanocarpa* have led to investigating the bioactives present in its extracts (12, 13). *A. melanocarpa* juice (200 mL) was shown to have a potent effect on postprandial glucose in healthy subjects after an oral meal tolerance test (14). This effect was imparted regardless of the gender of the subjects and was additionally shown to reduce the activity of dipeptidyl peptidase IV, α-glucosidase, and angiotensin-converting enzyme (ACE) in a dose-dependent manner (14). Table 1 summarizes studies which have successfully demonstrated the hypoglycemic, hypolipidemic, and antioxidant effects of various parts and extracts of the plant. Table 2 summarizes the studies dedicated to identifying the components and bioactive compounds of interest along with their findings. Furthermore, reviews such as those by Jurikova and others (15), Chrubasik et al. (10), Kokotkiewicz et al. (11), and Parzonko and Naruszewicz (16) have appeared recently highlighting the antidiabetic effects of *A. melanocarpa* in relation to mitigating hyperglycemia-induced oxidative stress and other precipitating conditions of the disease.

**Table 1:** Studies demonstrating the therapeutic properties of *Aronia melanocarpa*.

Administered component	Study model	Observed effects	Reference
Extract from *A. melanocarpa* leaves	Streptozotocin (STZ)-induced diabetic rats	Hypoglycemia	(17, 18)

*A. melanocarpa* fruit juice	STZ-induced diabetic rats	Hypoglycemia and hypolipidemia	(19)
		Reduced thiobarbituric acid (TBARS) concentration in blood, positive changes on total cholesterol and triglycerides, as well as attenuated kidneys’ hypertrophy and lipid peroxidation	(20)

	C57BL/6JmsSlc and KK-Ay mice	Significantly reduced body weight, weights of white adipose tissues and blood glucose level in KK-Ay mice given *A. melanocarpa* juice, along with reduced α-glucosidase activity in the upper portion of the small intestine	(21)

	C57BL/6J mice fed with a low-fat, high-sucrose, or high-fat (55% energy from fat) diet	Except for the significant reduction on body weight (especially for LFHS diet) no significant effect was found on adipose tissue gene expression, plasma insulin or triglycerides	(22)

	Healthy rats fed a maize starch (C) or high-carbohydrate, high-fat diet (H)	A significant reduction in visceral adiposity index, total body fat mass, and systolic blood pressure; improved glucose tolerance, liver, and cardiovascular structure and function (decreased total cholesterol and triglycerides) along with decreased macrovascular steatosis and portal inflammation	(23)

	Clinical study in type 2 diabetic patients	Reduced fasting blood glucose levels	(24, 25)

	Clinical study on healthy subjects	After a 3-week consumption, significant increase in serum antioxidant capacity was found (already at week 1), no change in blood lipid status, but reduced triglycerides	(26)

	Clinical study on healthy subjects	200 ml of Figure S2 in Supplementary Material summarizes all the disease conditions for which *A. melanocarpa* has been used to date and has been proven effective. juice reduced the postprandial blood glucose after an oral meal tolerance test and significantly reduced the activity of dipeptidyl peptidase IV, α-glucosidase and angiotensin-converting enzyme in a dose-dependent manner	(14)

Anthocyanin-rich fraction separated from *A. melanocarpa* fruit	Pancreatic β-cells	Scavenging effect of intracellular reactive oxygen species (ROS)	(27)
	Human HepG2 cells	Reduced ROS levels induced by high glucose	(28)

Anthocyanins and procyanidin-rich fraction from *A. melanocarpa*	*In vitro* evaluation	Reduced blood glucose levels due to inhibition of α-glucosidase	(29)
*A. melanocarpa* plant extract		A good source of phenolic compounds as compared with the other species examined in the study, which led to its identification as a potential functional food against diseases related to elevated oxidative stress levels	(30)

*A. melanocarpa* fruit extract	Healthy rats fed on normal diet	A decrease in the oxidative stress markers such as total antioxidant capacity, total thiol groups and glutathione; the enzymes CAT and ceruloplasmin were unaffected by the treatment	(17)

	Healthy rats fed with a high-fructose diet	A significant reduction in weight gain, epididymal fat accumulation, blood glucose and lipid metabolism (total and LDL cholesterol, triglycerides), increased plasma adiponectin levels, and decreased plasma TNF-α and IL-6 levels, along with gene expression activity in multiple pathways involved in insulin signaling, adipogenesis, and inflammation	(31)

	STZ-induced diabetic rats	A significantly lower inflammatory cytokines (TNF-α and IFN-γ) regardless of the concentration of *A. melanocarpa* extract that was administered	(32)

**Table 2:** Compositional details and bioactive compounds in *Aronia melanocarpa* fruits.

Reference	Components
(11, 25)	Sugar (10–18%), pectins (0.6–0.7%), sorbitol, parasorboside and small amount of fat (0.14% fresh weight, composed mainly of linoleic acid glycerides and phosphatidylinositol), minerals—K, Zn, Na, Ca, Mg, and Fe, vitamin B complex, vitamin C and A, and carotenoids, tannins, and triterpenes (b-sitosterol and campesterol), amygdalin, volatile compounds—benzaldehyde cyanohydrin, hydrocyanic acid, and benzaldehyde

(15)	Neochlorogenic and chlorogenic acids, cyanidin-3-galactoside, cyanidin-3-arabinoside, (−)-epicatechin units

(10, 11, 16, 33)	Procyanidins, anthocyanins, and phenolic acids

(34, 35)	Phenolic compounds, chlorogenic acid, neochlorogenic acid, and cyanidin-3-galactoside

(36, 37)	Procyanidins

(3, 9, 16)	(−)-epicatechin

## The Potential of *A. melanocarpa* in the Modulation of Other Precipitating Conditions of Diabetes

Although the number of published studies on *A. melanocarpa* increases by day, only a few have been conducted to evaluate its therapeutic effects clinically. According to Chrubasik et al. (10), only 13 clinical trials which encompassed various *A. melanocarpa* products for treatment of metabolic syndrome, hypercholesterolemia, and type 2 diabetes have been published to date, while 2 studies had been conducted on healthy participants, and another 3 on other health issues. Nevertheless, all studies showed significant improvements in the observed parameters at a clinical level. The initial use of *A. melanocarpa* by Native Americans was for the treatment of colds, but its popularity increased after it was introduced to Russia and Eastern Europe where it was extensively used as an anti-hypertensive drug (11). The anti-hypertensive potential of *A. melanocarpa* was proven by Kardum et al. (38). They conducted a 4-week intervention study with 200 mL of *A. melanocarpa* berry juice administered per day to subjects with pharmacologically untreated high normal blood pressure (BP) and grade I hypertension. In this study, the average 24-h and awake systolic (SBP) and diastolic BP (DBP) had significantly decreased, while these were higher in the group with a prevalence of sympathetic activity. Interestingly, reduction in SBP and DBP was more significant when a period of regular consumption of *A. melanocarpa* berry juice was followed (39). Another study by Broncel et al. (40) also demonstrated significant reductions in both SBP and DBP after 2 months of consumption of 300 mg of *A. melanocarpa* berry extract per day among patients with metabolic syndrome. Overall, these studies showed the potential of long-term consumption of *A. melanocarpa* berry juice, although periods of continuous usage were recommended to be accompanied with a period of abstain.

The therapeutic potential of *A. melanocarpa* was proven to be higher among people with increased cardiovascular risk (41), inferring that they should be one of the target populations to whom this plant extract should be administered. One of the underlying therapeutic mechanisms of action of *A. melanocarpa* is the stimulation of the endothelial formation of nitric oxide (NO) in coronary arteries (*via* phosphorilation of eNOS) (42). Yamane and others (43) showed that in spontaneously hypertensive rats, a diet containing freeze-dried *A. melanocarpa* berries significantly reduces SBP along with a significantly reduced ACE activity at 4 weeks. Inhibition of ACE activity has been attributed to anthocyanidins and flavonoids which are all highly abundant in *A. melanocarpa* (36, 37, 44, 45). More recently, the study by Bhaswant et al. (23) found similar beneficial effects on parameters related to the metabolic syndrome in rats fed with either *A. melanocarpa* juice or purple maize flour. The main conclusion of this study was that anthocyanins are the most probable bioactive components responsible for the observed beneficial effects in *A. melanocarpa*.

Another therapeutic potential of *A. melanocarpa* is its modulation of the lipoprotein profile (10, 11). This was particularly demonstrated in the study by Kardum and others (46) where significant reductions in the triglyceride content (TG) was observed among mildly hypertensive patients after 4 weeks of consumption of *A. melanocarpa* berry juice. Significant reduction in TG was also demonstrated in the study by Nowak and others (26), which was conducted in healthy individuals. In this study, men with mild hypercholesterolemia consumed 250 mL of the fruit juice for 3 weeks and achieved significant metabolic changes (40). It is important to note that regular consumption of *A. melanocarpa* is recommended even for people already on statin therapy (47).

The anti-inflammatory potential of *A. melanocarpa* juice was demonstrated in few studies through reduced levels of cytokines (38, 40), as well as in studies which used patients with cardiovascular disease (47). Badescu and others (32) demonstrated that the chronic inflammatory reaction related to diabetes mellitus improves under the action of polyphenols from *A. melanocarpa*, specifically through its ability to lower TNF-α and IFN-γ.

## Conclusion and Future Directions

At present, approximately 415 million people around the world have been contracted with diabetes, while a constant increase in the number is expected. Furthermore, pre-diabetes (defined as impaired fasting glucose or impaired glucose tolerance) has observed to significantly increase the risk for developing diabetes and its complications, where the current prevalence is estimated to be 6.7% (48). The quality of life is affected by this disease while the life expectancy among diabetics is reduced by 5–10 years as compared with the healthy population, with cardiovascular complications representing the major cause of mortality. The financial burden occurring from this disease is evident to be substantial. Globally, 12% of the total health-care resources are being spent for diabetes treatment, ranging from 5 to 20% (48). However, along with reduced financial allocations toward health care, especially in terms of preventive measures and pharmaceuticals, the burden falls onto patients and their families. People with diabetes spend 2.5 times for health care from their own financial reserves than their healthy counterparts (49), especially if they have developed cardiovascular disease (50). Finding a way to bridge the gap between current treatments, preventive measures (directed toward pre-diabetes as well as delay of diabetes complications), possible adjuvant therapies, and dietary and lifestyle modification is strongly encouraged given this situation (48). As highlighted in this review, *A. melanocarpa* berry juice and plant extract has displayed evidence as a potent modulator of hyperglycemia-related oxidative stress which is directly correlated with its complications, in particular, cardiovascular disease. Nevertheless, continuous use of *A. melanocarpa* is recommended to be accompanied with the same period of abstain. Also, people with increased cardiovascular risk (i.e., with abdominal obesity, mild hypercholesterolemia, grade I hypertension) seem to benefit more from the consumption of *A. melanocarpa* berry juice and extract. Thus, overall, consumption of *A. melanocarpa* could be recommended as a possible approach to reducing the financial burden for both diabetics and their families, as well as that of national health-care systems in countries where diabetes poses a significant liability. Additionally, the fruit and its extract appear to have a multitude of beneficial effects against other disease conditions, which could potentially be explored and scientifically substantiated through systematic studies and investigations.

## Author Contributions

IB, AM, KŠ, SJ, MM, HZ, and VW equally contributed to the acquisition of information, drafting the manuscript, and approving the final version.

## Conflict of Interest Statement

The authors declare that the research was conducted in the absence of any commercial or financial relationships that could be construed as a potential conflict of interest.

## Supplementary Material

Figure S1 Disease conditions for which administration/consumption of *A. melanocarpa* has proven to be effective.

Figure S2 Images of **(A)** *A. melanocarpa* in fields during summer-time **(B)**, bushes with fruits, **(C)** fruits & leaves and **(D)** the chemical structures of some of the potent bioactive compounds present in *A. melanocarpa*.
