# Supplementary figures and images for: Antidiabetic Effects of Aronia melanocarpa and Its Other Therapeutic Properties
Source: Front Nutr. 2017 Nov 6;4:53. doi: 10.3389/fnut.2017.00053 (PMC5681526; doi:10.3389/fnut.2017.00053)

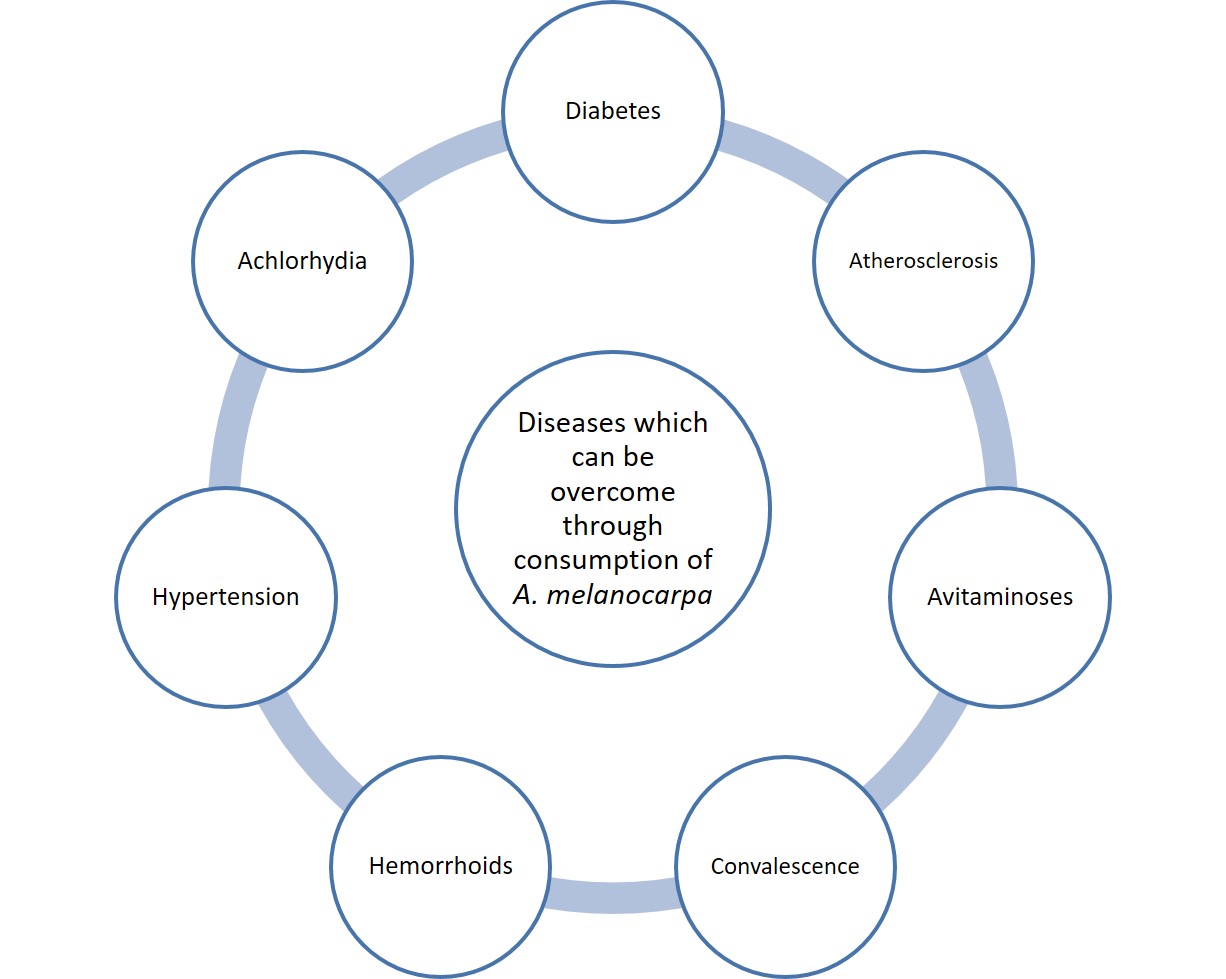

Supplement: Figure S1 — Disease conditions for which administration/consumption of A. melanocarpa has proven to be effective. [file Image_1.JPEG]

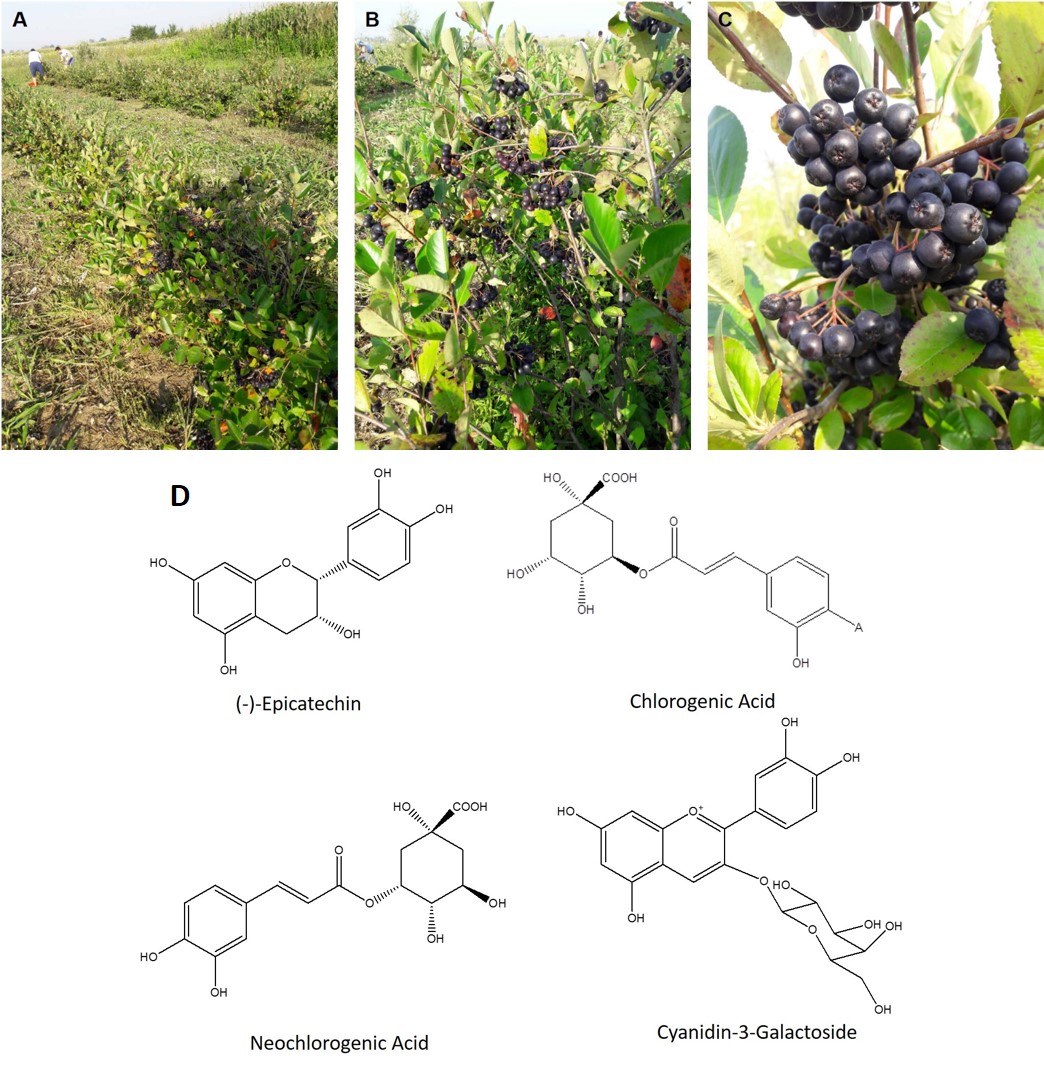

Supplement: Figure S2 — Images of (A) A. melanocarpa in fields during summer-time (B), bushes with fruits, (C) fruits & leaves and (D) the chemical structures of some of the potent bioactive compounds present in A. melanocarpa. [file Image_2.JPEG]
